# Systematic review and meta-analysis of dairy cow responses to rumen-protected methionine supplementation before and after calving

**DOI:** 10.3168/jdsc.2023-0512

**Published:** 2024-03-02

**Authors:** G.I. Zanton, M.Z. Toledo

**Affiliations:** 1 USDA–Agricultural Research Service, US Dairy Forage Research Center, Madison, WI 53706; 2 Department of Animal and Dairy Sciences, University of Wisconsin–Madison, Madison, WI 53706

## Abstract

Balancing dairy cow diets for AA is an effective strategy to reduce dietary CP concentration, maintain levels of productivity, and increase nitrogen use efficiency. Most studies evaluating supplemental rumen-protected Met (sRPMet) focus on cows in established lactation; however, there is an increasing body of evidence suggesting that initiating sRPMet feeding to transition dairy cows is beneficial to production, reproduction, and health. Therefore, the objective of this study was to evaluate the effects of feeding sRPMet before and after calving through meta-analysis on pre- and postpartum performance and selected metabolic parameter responses. A literature search was conducted for published papers reporting on the effects of feeding sRPMet starting before parturition and continuing through early lactation, resulting in 21 publications with 40 treatment comparisons. Studies provided sRPMet both before (average of 8.20 [±2.94 SD] g of metabolizable sRPMet/d, which began at 19.3 [±4.23 SD] d before calving) and after calving (10.53 [±3.30 SD] g of metabolizable sRPMet/d for an average of 85.9 [±38.36 SD] DIM). Prepartum DMI and pre- and postpartum BW and BCS were unaffected by sRPMet. In contrast, postpartum DMI, milk yield, milk fat and true protein yield, and milk fat and true protein concentration were increased by sRPMet. Most production responses to sRPMet declined as lactation progressed where the predicted response in milk fat and true protein yield was 118 and 92 g/d at 21 DIM, respectively. Postpartum circulating metabolites were unaffected by sRPMet; however, the sample sizes for these analyses were much lower than for production responses. This meta-analysis indicates that feeding sRPMet before and after calving results in increased productivity beyond that which would be expected by providing sRPMet in established lactation alone.

## Highlights

• Transition cow sRPMet did not affect prepartum responses.
• Postpartum intake, production, and component concentration increased from sRPMet.
• Milk fat and true protein increased by 118 and 92 g/day at 21 days in milk, respectively.
• Several responses to sRPMet declined with increasing days in milk.

## Graphical Abstract

**Figure:**
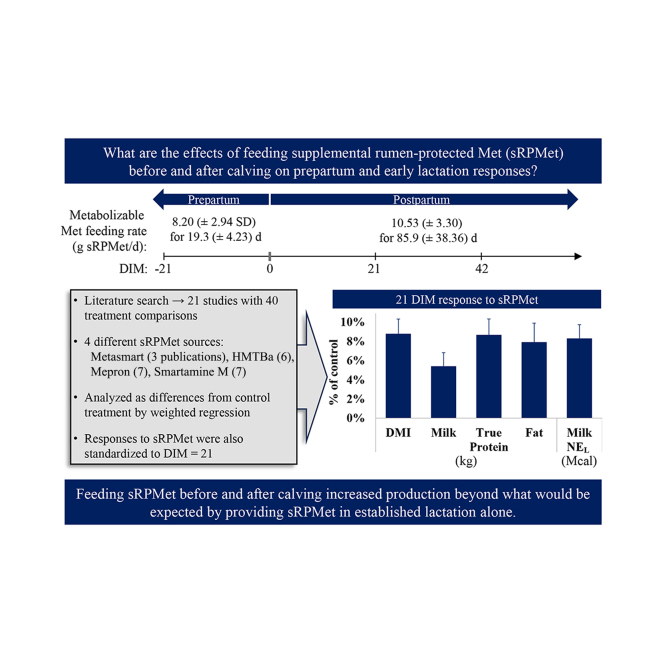
**Summary:** Meeting the protein requirement of dairy cows is achieved by providing an adequate quantity of amino acids in appropriate proportions for maintaining health and productivity across lactation. Methionine is an essential amino acid that has many physiological functions beyond its role in the synthesis of milk protein. Supplementing rumen-protected Met (sRPMet) in established lactation results in increased production of milk protein and inconsistent increases in milk fat. Feeding sRPMet to dairy cows both before and after calving has been evaluated in several experiments that are summarized through meta-analysis in this study. Providing sRPMet to dairy cows before and after calving resulted in increased dry matter intake, milk yield, milk fat and protein yield, and milk fat and protein concentration. Several of these production responses to sRPMet declined as lactation progressed, indicating that feeding sRPMet before and after calving increased production beyond what would be expected by providing sRPMet in established lactation alone.

Dairy cow protein utilization and requirements change throughout lactation (Letelier et al., 2022) and can be especially high in very early lactation (Wu and Satter, 2000; Larsen et al., 2014). During the peripartum period through early lactation, many cows enter a period of overt negative energy and protein balance (Bell et al., 2000) that may affect nutritional requirements for optimal production and health. While prepartum protein supplementation could potentially affect postpartum protein metabolism, experimental results have shown limited beneficial productive responses (Doepel et al., 2002; Zang et al., 2022). Specific AA are required for many nonprotein functions and may also hold great importance around parturition in dairy cows (Wu et al., 2014). One AA that has been studied extensively during established lactation and the peripartum period is Met (Socha et al., 2008; Osorio et al., 2013; Zanton et al., 2014). Although the effects of Met in established lactation have been thoroughly analyzed in previous studies (Patton, 2010; Robinson, 2010; Zanton et al., 2014), the quantitative effects of initiating supplemental rumen-protected Met (**sRPMet**) feeding before calving are less clear. Therefore, the objective of this meta-analysis was to evaluate the effects of feeding sRPMet before and after calving on early lactation production responses and selected metabolites in postpartum, transition dairy cows. Our hypothesis was that feeding sRPMet around calving would increase productive performance.

Because this study was based on previously published literature and did not use animals in any way, no Institutional Animal Care and Use Committee protocol was required. Literature search was conducted in Google Scholar, Scopus, and Web of Science (using the search terms “rumen protected” AND methionine AND cow AND transition OR calving OR partum) and in reference lists for published papers reporting on the effects of Met supplementation starting before parturition and continuing through early lactation. To be eligible for this analysis, studies had to report the source and level of sRPMet, individual feeding of sRPMet had to begin before calving and continue into early lactation, and studies had to be published in a peer-reviewed journal. This search resulted in final dataset including 21 publications (Polan et al., 1970; Holter et al., 1972; Bhargava et al., 1977; Lundquist et al., 1985; Overton et al., 1996; Phillips et al., 2003; Piepenbrink et al., 2004; Socha et al., 2005; Kudrna et al., 2009; Ordway et al., 2009; Preynat et al., 2009; Strzetelski et al., 2009; Ardalan et al., 2010; Osorio et al., 2013; Sun et al., 2016; Zhou et al., 2016; Batistel et al., 2017; Cetin et al., 2018; Tamura et al., 2019; Potts et al., 2020; Cardoso et al., 2021) with 40 treatment comparisons (sRPMet vs. control). The study of Girard et al. (2005) was identified in the search but not included in the analysis due to the postpartum feeding period including the complete lactation instead of predominantly early lactation. The study of Toledo et al. (2021) was excluded from this analysis because pen was the experimental unit. However, the responses of this study are included in the graphical representation of the results as a reference, large-scale study conducted under industry-relevant conditions (the number of individual cows exceeded the next largest study by 5-fold). In all studies identified except for in 1 study where a main factor of prepartum diets excluded sRPMet (Kudrna et al., 2009), sRPMet was included in both the prepartum and postpartum diets. Four different Met sources were fed to cows in the studies entering this analysis: Metasmart in 3 papers; 2-hydroxy-4-methylthio butanoic acid (**HMTBa**) in 6 papers; Mepron in 7 papers; and Smartamine in 7 papers, including 2 papers comparing 2 sources (Smartamine and Metasmart: Ordway et al., 2009; Osorio et al., 2013). For each of these sources, published values were used to calculate metabolizable Met (**MPMet**) from total sRPMet intake because MPMet of the rumen-protected sources was not experimentally determined in most studies (Zanton et al., 2014). Thus, 40% (Koenig et al., 2002), 50% (Graulet et al., 2005), 75% (Overton et al., 1996), and 80% (Schwab, 1995) of Met intake contributed to MPMet for HMTBa, Metasmart, Mepron, and Smartamine, respectively, although more robust response estimates would result from independent determination within the context of each study. Design variables captured from the papers were author and year of publication, source of sRPMet, level of choline supplementation, DIM at the start of supplementation (represented as negative values from parturition), DIM at the end of the study, type of feeding (top-dressed vs. mixed into the diet), lactation number, pre- and postpartum supplementation rate, and pre- and postpartum dietary CP and NDF. Outcome variables captured from the papers were pre- and postpartum DMI; yield of milk, fat, protein, and lactose; concentration of fat, protein, and lactose; MUN; cow BW (prepartum, calving, and postpartum); pre- and postpartum nonesterified fatty acids (**NEFA**); BHB; glucose plasma concentration; and transition cow metabolic disorder incidence numbers. Indicators of variability (SE or SD), n per treatment, and units of expression were also captured. Milk megacalorie output was calculated as described in NRC (2001).

Initially, a mixed effect analysis (St-Pierre, 2001) was pursued with a continuous level of sRPMet as the fixed, independent variable (using 0 g/d sRPMet intake for control treatments) and study as the random effect. However, pre- and postpartum supplementation levels within study (g/d or concentration) were not consistent in most studies. Therefore, within study outcome variables were expressed as the difference between sRPMet value and control value (i.e., milk yield response = sRPMet milk yield − control milk yield) and coded as a unique experiment based on each unique control diet (i.e., a paper reporting results of a 2 × 2 factorial with and without Met and choline would be coded as 2 experiments based on 2 levels of choline in the Met-supplemented treatments; Sun et al., 2016). These responses were analyzed in SAS v9.4 (SAS Institute Inc., 2013) using a fixed effects model where variance associated with the overall effects of sRPMet and design covariates were factors of interest. Of the design variables captured, there was a sufficient level of reporting such that fixed effects of ending DIM, source of sRPMet, and level of postpartum sRPMet were evaluated as covariates and these will be discussed when significant (*P* ≤ 0.05). Due to the variability in the duration of lactation follow-up measurements, when ending DIM covariate was significant (*P* ≤ 0.05), response to sRPMet was also estimated at DIM = 21 in addition to the point estimate of the overall effect of sRPMet, which is estimated at the average of ending DIM. Responses were weighted by the square root of the number of experimental units (√n) in the treatment group (Lean et al., 2009). We used √n as the weight as opposed to other weighting factors such as the inverse of the variance (St-Pierre, 2001) or the trimmed inverse of the SEM processed independently for mixed and fixed effects models (Roman-Garcia et al., 2016) for several reasons. First, √n scales directly with the inverse of SEM with lesser requirements for trimming because it is not divided by the SD. Additionally, √n is independent of modeling decisions (mixed effects vs. fixed effects, repeated measures, covariates, and so on) in the source statistical analysis. Finally, for several papers or responses, and especially with calculated responses (i.e., milk Mcal output), SEM was unavailable whereas √n was available for all responses and all studies. Residual diagnostics (|studentized residual| > 3) resulted in removal of some observations (Polan et al., 1970: 0.8% HMTBa for production responses and all treatments for NEFA; Potts et al., 2020: multiparous, rumen-protected choline; Strzetelski et al., 2009: 30 g of Smartamine for postpartum glucose concentration).

As shown in Table 1, initiating sRPMet feeding prepartum did not affect prepartum DMI, BW, or BCS (*P* > 0.15). Average level of supplementation before calving was 8.20 (±2.94 SD) g/d of MPMet, which began at 19.3 (±4.23 SD) d prepartum on average, leaving a relatively short duration for observing changes in these responses over this period. In contrast after calving, cows were supplemented with an average of 10.53 (±3.30 SD) g/d of MPMet for an average of 85.9 (±38.36 SD) DIM. While this level of postpartum supplementation is lower than that typically fed in studies conducted with cows in established lactation (Zanton et al., 2014), production responses in established lactation also increase with increasing level of sRPMet. This may indicate that changing the feeding rate of sRPMet postpartum would result in different responses in postpartum production than the magnitude of responses observed in this analysis. However, within the range of sRPMet fed in these studies, covariate analyses (results not shown) did not result in a significant relationship between production and MPMet from sRPMet. Future studies in the postpartum period conducted as a dose titration may be warranted.

**Table 1:** Responses to initiating supplemental rumen-protected Met (sRPMet) feeding to transition cows^1^

Item	Control				Response to sRPMet				
	N^2^	n^2^	Mean	SD	N^2^	n^2^	Mean	SEM	*P*-value
Prepartum^3^									
DMI, kg/d	22	309	13.1	1.68	26	362	0.19	0.140	0.184
BW, kg	15	221	713	57.4	19	274	−0.08	2.40	0.974
BCS	14	207	3.51	0.231	18	260	−0.01	0.020	0.846
Postpartum^4^									
DMI,^5^ kg/d	29	387	19.4	3.54	40	510	0.45	0.156	0.006
DMI_21DMI_							1.38	0.283	<0.001
BW, kg	21	303	620	40.9	29	404	−2.13	3.10	0.498
BCS	16	238	2.92	0.326	20	291	0.01	0.031	0.707
Yield									
Milk,^5^ kg/d	29	387	35.6	6.44	40	510	0.80	0.271	0.006
Milk_21DIM_							2.13	0.515	<0.001
Fat,^5^ g/d	29	387	1,288	285.8	40	510	75.8	11.63	<0.001
Fat_21DIM_							117.6	23.32	<0.001
True protein,^5^ g/d	26	362	1,032	168.8	34	456	43.4	10.4	<0.001
True protein_21DIM_							92.1	18.39	<0.001
Concentration, %									
Fat	29	387	3.62	0.303	40	510	0.150	0.032	<0.001
True protein^5^, ^6^	26	362	2.85	0.094	34	456	0.066	0.016	<0.001
True protein_21DIM_							0.140	0.028	<0.001
Mcal secreted^7^									
/d^5^	26	362	24.94	4.64	34	456	1.13	0.211	<0.001
/d_21DIM_							2.18	0.363	<0.001
/kg DMI	26	362	1.30	0.235	34	456	0.015	0.010	0.126

1 Control and response estimates weighted by the √n, where n is the number of cows for control or sRPMet groups.

2 N = number of control means or sRPMet responses; n = number of control or sRPMet cows.

3 Length of prepartum sRPMet feeding averaged 19.3 d (±4.23 SD) with 8.20 g (±2.94 SD) of metabolizable Met.

4 Length of postpartum observations averaged 85.9 d (±38.36 SD) with 10.53 g (±3.30 SD) of metabolizable Met.

5 Dependent on the duration of measurement (final DIM *P* < 0.05).

6 Source effect (*P* = 0.013) resulting from no response due to HMTBa supplementation (*P* > 0.60) and a positive response from all other sources (0.101% ± 0.018% increase; *P* < 0.001). No other differential source effects were observed.

7 Calculated according to the NRC (2001).

After calving, average BW and BCS were unaffected by sRPMet, but DMI, milk yield, milk fat yield and concentration, and milk protein yield and concentration were all increased in the sRPMet group (Table 1). Consistent with results reported elsewhere on stage of lactation (Schwab et al., 1992; Socha et al., 2008), ending DIM affected the magnitude of these responses wherein the response to sRPMet was maximized in earlier lactation compared with a diminishing response when ending DIM increased (regression results and responses for individual studies are shown in Figure 1). In an attempt to evaluate the production effects at the conclusion of the transition period and to standardize the comparison of responses, ending DIM was included as a fixed effect continuous predictor, which was then used to estimate the sRPMet response at 21 DIM. When estimated at 21 DIM, sRPMet pre- and postpartum resulted in 1.38 (±0.283) kg/d additional DMI, 2.13 (±0.515) kg/d additional milk, 118 (±23) g/d additional milk fat, and 92 (±18) g/d additional milk true protein, where responses in milk fat and true protein resulted from concomitant increases in both milk yield and component concentrations. These production responses were not accompanied by changes in circulating energy metabolite concentrations (response to sRPMet in plasma NEFA = −17.3 [±19.49] m*M*, BHB = −0.053 [±0.039] m*M*, and glucose = −0.017 [±0.022] m*M*, all *P* > 0.15 and n < 20). However, a broader consideration of the potential effects on health and transition disorders is needed in subsequent research due to the limited and inconsistently reported results in these production papers. There is no consensus on the Met bioavailability of HMTBa (NASEM, 2021) and the remaining sRPMet source bioavailability values were taken from literature values and not evaluated directly under the conditions of these experiments. To address potential source effects, we included a categorical effect of sRPMet source on the response variables. The only sRPMet source effect observed was for milk true protein concentration, in which cows fed HMTBa did not have a milk protein concentration different from the control cows, whereas a positive response was detected across all other sources for milk protein concentration (0.101% ± 0.018% increase; *P* < 0.001). No other differential source effects on responses were observed.

**Figure 1:**
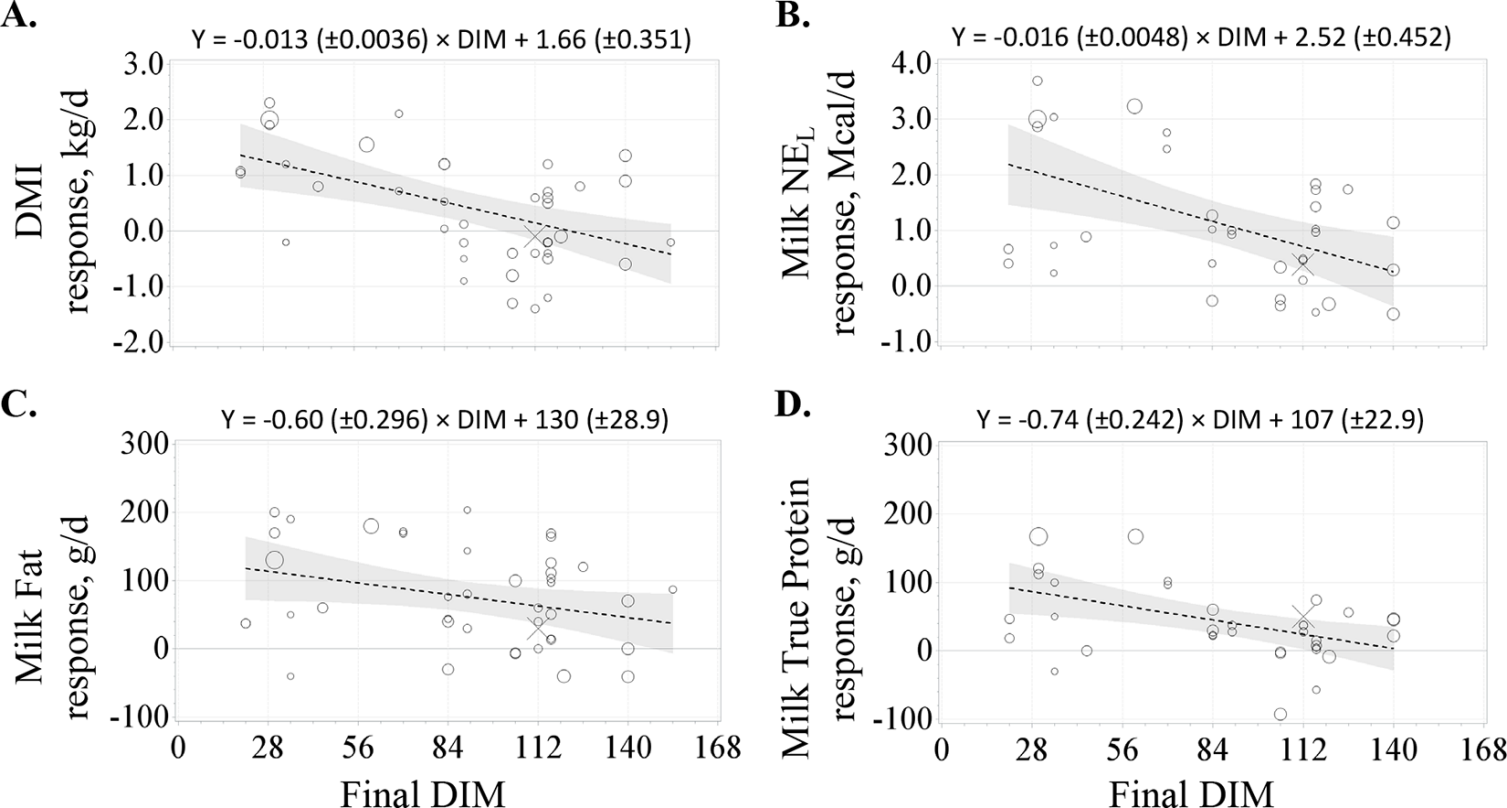
Within-study responses to supplemental prepartum and postpartum rumen-protected Met (sRPMet) decline with final study DIM in (A) DMI (kg/d), (B) milk NE_L_ output (Mcal/d; NRC, 2001), (C) milk fat yield (g/d), and (D) milk true protein yield (g/d). Positive values indicate that the response was greater in cows with sRPMet than the cows in the control groups. The size of the circle symbols represents the weight (square root of the number of experimental units fed Met) that each treatment mean received during analysis. The value X is the response to Met over control in the study of Toledo et al. (2021). That study was not included in this analysis due to differences from the other studies entering the analysis since pen was the experimental unit and the number of individual cows (observational units) exceeded the next largest study by 5-fold. However, the responses of this study are included as a reference, large-scale study conducted under industry-relevant conditions. The best-fit linear regression equation is represented by dashed lines surrounded by the shaded 95% CI.

The results of this meta-analysis align closely with the large-scale, pen-feeding study reported by Toledo et al. (2021) for DMI, milk NE_L_ output, milk fat yield, and milk true protein yield (Figure 1). The scale of that experiment, the independence from model parameterization in this meta-analysis, and the consistency with the current results improve the confidence in the results of the meta-analysis derived from studies across time, sRPMet sources, and study sites. The magnitude of the production responses to pre- and postpartum sRPMet estimated at 21 DIM is considerably greater than the responses estimated in established lactation. For example, Zanton et al. (2014) estimated that the response to sRPMet in established lactation ranged between −0.25 to 0.31 kg/d for DMI, −0.34 to 0.31 kg/d for milk yield, 6 to 45 g/d for milk fat, and 13 to 35 g/d for milk protein yield, depending on the source of sRPMet. Additionally, in the analysis of Zanton et al. (2014), for each additional gram of MPMet fed in established lactation, 2.23 g/d of milk true protein was secreted across sources, which would predict an additional 23.48 g/d of milk true protein based on average postpartum supplementation for studies in this analysis. In contrast, when sRPMet feeding was begun prepartum, the overall predicted milk protein yield was approximately double this amount (43 g/d) and the 21 DIM estimate was 92 g/d. The milk true protein yield was not predicted to decline to 22.48 g/d until 113 DIM. While milk fat yield varied according to source in Zanton et al. (2014), using the milk fat response to sRPMet for sources other than HMTBa of 1.9 g milk fat/g of additional MPMet resulted in a predicted milk fat yield of 20.00 g/d, whereas using the value for HMTBa (5.38 g milk fat yield/g of additional MPMet) would predict 56.65 g milk fat/d on average. This is less than the milk fat yield observed in this analysis when sRPMet feeding began before calving of 76 g/d overall and 118 g/d when estimated at 21 DIM.

These comparisons with studies in established lactation indicate that the production response to sRPMet is greatest in the immediate postpartum period. The reason for this difference cannot be directly determined from this analysis but may be indicated by a recent report by Toledo et al. (2023) in which feeding sRPMet lessened the negative effects of health disorders on milk protein production and time to pregnancy. This resulted from an interaction of feeding sRPMet with health status in which cows with metabolic disorders responded most strongly to sRPMet. Whether these responses are differentially affected due to prepartum versus postpartum supplementation is unknown. Since most studies fed sRPMet during both the prepartum and postpartum transition period and then, in many studies, further into lactation, ascribing the response to any of these specific periods is impossible due to this confounding. Kudrna et al. (2009) fed diets either with or without sRPMet pre- or postpartum in a 2 × 2 factorial arrangement of treatments and was unable to determine a significant additive or interactive response between pre- or postpartum supplementation. The lack of clarity on the effects of feeding sRPMet in transition dairy cows during either the pre- or postpartum transition period leads us to conclude that maximal productive responses were obtained when feeding sRPMet during both the pre- and postpartum transition period. However, the optimal economical approach to feeding sRPMet is unknown and will require further research to clarify the appropriate time, dose, and conditions to optimize the response to sRPMet for transition dairy cows.
